# Invisible minority stress and addictive behaviors: disparities in psychological distress and e-cigarette use among lesbian, gay, and bisexual adults in China

**DOI:** 10.1186/s40359-025-03875-7

**Published:** 2026-01-17

**Authors:** Luxi Zhang, Sha Sarah Qiu, Xinshu Zhao, Song Harris Ao, Zijun Chloe Wang

**Affiliations:** 1 Department of Communication, Faculty of Social Science, University of Macau Avenida da Universidade, Humanities & Social Sciences Building (E21B), Macau, China; 2 School of Journalism and Communication, Sun Yat-sen University, Guangzhou, China

**Keywords:** LGB, Sexual minority, Psychological distress, E-cigarette use, Addiction, Health psychology

## Abstract

**Background:**

Evidence shows that lesbian, gay, and bisexual (LGB) groups face disparities in e-cigarette use, but little is known about e-cigarette use among the Chinese LGB population. China, with the largest LGB population and as the leading global producer in the e-cigarette industry, presents a unique context for studying this phenomenon.

**Methods:**

Using the theoretical framework of minority stress, this study hypothesized a moderated mediation model to test the mediating effect of psychological distress and the moderating effect of eHealth use on the association between sexual orientation and e-cigarette use. Data from a nationally representative Chinese survey conducted between January and September 2023 (*n* = 4,901) were analyzed.

**Results:**

The results indicated that LGB individuals had 11.1% higher odds of distress compared to heterosexual populations, was associated with higher e-cigarette use (*bp* = 0.038, *p* < .001). However, the direct association between sexual minority identity and e-cigarette use was non-significant. Furthermore, eHealth use moderated the disparities in psychological distress between LGB and heterosexual groups (*bp* = − 0.160, *p* < .05).

**Conclusion:**

The pattern of e-cigarette use among Chinese sexual minorities is distinct: e-cigarettes are less commercialized and serve mainly as a coping mechanism. These findings highlight the importance of mental health support and eHealth interventions in reducing psychological distress and e-cigarette use among LGB individuals in China.

**Supplementary Information:**

The online version contains supplementary material available at 10.1186/s40359-025-03875-7.

## Background

China has the largest lesbian, gay, and bisexual (LGB) population globally [1]. Raising offspring is a fundamental aspect of Chinese culture. Contrary to dominant cultural values, LGB individuals in China constitute an invisible minority under significant social pressure [1, 2]. For instance, a survey by the United Nations Development Programme and Beijing LGBT Center found that 17%−25% of heterosexual participants did not accept LGB children [1]. Additionally, LGB groups experience health disparities and inequalities. Research indicates that LGB individuals are more likely to suffer psychological distress and engage in risky behaviors compared to heterosexual populations, due to social discrimination, minority stress, and historical stigmatization [3–7].

The use of electronic cigarettes (e-cigarettes) has emerged as a new form of smoking in recent decades, raising public health concerns [8]. China is the world’s largest manufacture of e-cigarettes, producing approximately 90% of e-cigarettes globally [9]. However, the prevalence of e-cigarette use in China remains relatively low compared with Western countries. National surveys reported that fewer than 2% of Chinese adults were current e-cigarette users in recent years [10, 11], whereas about 10% of adults in the UK and 6.5% in the U.S. reported current use [12, 13]. However, the prevalence substantially increased from 2015 to 2019 [14]. Notably, the Chinese government has implemented a series of stringent regulations on e-cigarette marketing and sales since 2018 to protect minors [15]. For example, China banned sales to youth in 2018, online advertisements and sales in 2019, and implemented flavor bans in 2022 [15].

Besides minors, LGB individuals are another vulnerable group to e-cigarette use. Evidence shows that sexual minority populations are at higher risk of e-cigarette use compared to heterosexual populations [16–18]. The tobacco industry was among the first to identify and target LGB consumers [19, 20]. LGB groups are targeted by e-cigarette advertisements and report higher e-cigarette use [20]. For instance, the consumption rate of e-cigarettes in the U.S. is nearly twice as high among LGB adults as among heterosexual individuals [16–18]. While there are growing studies investigating health inequalities in mental health and e-cigarette use among sexual minority populations in Western countries, the situation in China remains underexplored. This study aims to comprehensively explore mental stress and e-cigarette use among LGB population in China.

The minority stress theory provides a framework for understanding psychological distress and risky behaviors among sexual minority populations. Derived from social and psychological theories on social interaction, stress, and stigma [21–23], Brooks (1981) first introduced the term “minority stress” to describe the relationship between mental stress and minority status [21]. Meyer (2003) expanded this concept to explain the long-term effects of social environment and interaction on sexual minority populations [24]. Sexual minority populations experience chronic, excessive stress related to their stigmatized identities in a culture that privileges heterosexuality. This prolonged stress can lead to mental health issues such as depression, anxiety, and mental disorders [24, 25]. Empirical studies within this framework have shown that LGB individuals have higher odds of psychological distress compared to their heterosexual counterparts [25–27].

Furthermore, psychological distress is often associated with risky behaviors [28–31]. These risky behaviors are the avoidance strategy to cope with undesirable emotions [29, 32]. The minority stress framework posits that higher rates of addictive behaviors, such as e-cigarette use, among LGB groups are driven by mental distress under minority stress [24, 33, 34], indicating a mediation effect of psychological distress. Empirical evidence consistently supports that psychological distress can mediate e-cigarette use [35, 36]. For instance, a longitudinal study demonstrated that mental distress mediates the association between social media use and subsequent e-cigarette use [37]. Building upon these previous findings, we propose the following hypotheses to investigate disparities in e-cigarette use between LGB and heterosexual populations and the underlying mechanisms:

- H1. Sexual orientation is directly associated with e-cigarette use, i.e., the LGB population reports more e-cigarettes use than the heterosexual population.
- H2. Sexual orientation is indirectly associated with e-cigarette use through psychological distress, i.e., the LGB population reports more e-cigarettes use due to elevated psychological distress.

The use of eHealth technologies has grown rapidly over recent decades, with abundant findings demonstrating its essential role in improving human health [38, 39]. For example, eHealth has been found to be effective in treating psychological distress [40, 41] and promoting smoking cessation [42, 43]. Particularly, recent studies have demonstrated that eHealth has a positive impact on the health outcomes of LGB population [44, 45]. The unique features of eHealth, such as accessibility, privacy protection, sense of community, and access to professional health knowledge [44, 46], create a safer environment for this vulnerable minority group. For instance, users can access resources anytime and anywhere without the constraints of clinic hours or geographic distance [47]. Anonymity in eHealth platforms reduces the psychological burden for vulnerable groups, allowing them to seek information or support more comfortably [48]. Online communities facilitate peer interactions, enhancing social support, while professional health information is readily available and can be updated more quickly than in conventional healthcare settings [49]. Together, these features create a safer and more supportive environment for sexual minority populations. An experiment testing the effect of eHealth among LGB adults showed that psychological distress of LGB can be reduced by using eHealth services [45].

In China, where internet users reached 1.079 billion by 2023 (CNNIC, 2023), digital healthcare reform has been actively promoted through the integration of health information and communication technologies (ICTs) (Lv et al., 2019; Wu et al., 2019). eHealth applications are widely adopted in public health, suggesting potential impacts on reducing mental distress among the LGB community. For instance, increased eHealth utilization may narrow the psychological distress gap between LGB and heterosexual individuals [44, 45]. However, there is limited evidence that eHealth use contributes to reducing e-cigarette use. As Holland (2017) emphasized [50], moderation hypotheses should be grounded in solid theoretical and empirical support. Guided by these considerations, we propose the following hypothesis:

- H3. eHealth use negatively moderates the association between sexual orientation and psychological distress, i.e., higher levels of eHealth predict a weaker association between being LGB and experiencing distress.

**Fig. 1:**
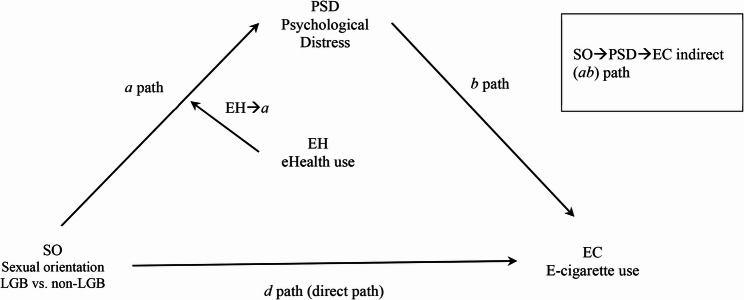
Conceptual model

Figure 1 depicts the conceptual model that integrates all the relationships above.

## Methods

### Data source and sample

Before commencing the study, the authors obtained research ethics approval from their institution. An anonymous email survey using Kantar (www.kantar.com) was conducted between January and September 2023. The survey included a random sample of 4,979 adults from 31 provinces (or equivalent administrative regions) in China, selected via quota sampling to match age and gender demographics based on the 2020 Chinese Seventh National Census (www.stats.gov.cn). Missing data, which amounted to less than 1.0% of the dataset, were excluded from regression analyses through listwise deletion.

### Measures

The dependent variable e-cigarette use measures the number of days the respondents used e-cigarette in the past 30 days (0–30) [51].

The independent variable sexual orientation was measured from an item asking the sexual orientation of the responders. Being homosexual (gay or lesbian) and bisexual were recoded as 1, being heterosexual or straight were recoded as 0.

The mediator psychological distress was measured using the validated Patient Health Questionnaire for Depression and Anxiety (PHQ-4) [52]. Psychological distress was the sum of four items (Cronbach’s α = 0.881) measuring the frequency the respondents experienced four symptoms of psychological distress in the past two weeks: (1) little interest or pleasure in doing things; (2) feeling down, depressed, or hopeless; (3) feeling nervous, anxious, or on edge; (4) not being able to stop or control worrying. Each item was rated on a four-point Likert scale (1 = “never” to 4 = “every day”). Each response was then linearly transformed to a 0–1 scale, and the four items were summed to create a composite score. The resulting psychological distress variable ranges from 0 to 4, where 0 indicates never experiencing any of the four symptoms and 4 indicates experiencing all four symptoms every day [53].

The moderator eHealth use was the sum of nine items that asked respondents whether they had engaged in nine kinds of eHealth activities, such as looking for health or medical information online; using Internet to communicate with a doctor; looking up medical test results online and buying medicines online. Each item was coded 0 or 1 where 0 indicating not conducting the kind of eHealth activity and 1 indicating performing the activity. The compound variable eHealth use ranges from 0 to 9 (KR-20 = 0.673), indicating 0 to 9 kinds of eHealth activities the respondents might have conducted. While the KR-20 coefficient for eHealth use was 0.673, it should be noted that this may represent a lower-bound estimate of reliability due to the binary nature of the items [54, 55]. Nevertheless, this construction method has been validated in previous empirical research [41, 56].

To reduce possible confounding, demographic variables age, gender, education, annual household income, and partnership were included as covariates in the regression analyses. See Table 1 for demographic details.

**Table 1:** Demographic features of respondents

Sexual Orientation (*n*. %)	
Homosexual, or gay or lesbian; Bisexual; Something else	149 (3.0)
Heterosexual, or straight	4,729 (96.4)
Age (18–70, Mean ± SD)	40.41 ± 13.97
Gender (n. %)	
Female	2,390 (48.8)
Male	2,511 (51.2)
Education (n. %)	
Primary school and below	42 (0.9)
Junior high school	182 (3.7)
High school	830 (16.9)
Vocation school	1,571 (32.1)
College and above	2,276 (46.4)
Annual household income (n. %)	
¥ 30,000 to ¥ 79,999	493 (10.1)
¥ 80,000 to ¥ 149,999	1,497 (30.5)
¥ 150,000 to ¥ 499,999	2,430 (49.6)
¥ 500,000 to ¥ 999,999	442 (9.0)
¥1,000,000 or more	39 (0.8)
Partnership (n. %)	
living with a romantic partner	3,994 (81.5)
living without a romantic partner	907 (18.5)
*N*	4,901

a SD stands for standard deviation

### Statistical analysis

Data analysis utilized SPSS (v26) and the SPSS macro PROCESS [57]. Initially, descriptive analyses were conducted for key variables, followed by Pearson correlation to examine their correlations. Subsequently, multivariate linear regressions were performed to investigate mediation and moderation effects. For mediation analysis, SPSS macro PROCESS model 4 [57] was employed to identify the mediating effects of psychological distress. Additionally, PROCESS model 7 was utilized to assess the moderating effects of eHealth use on the relationship between sexual orientation and psychological distress. To test standardized regression coefficients, we employed both standarlized *β* and percentage coefficient (*bp*). *Bp* is a b coefficient when independent variable and dependent varable are both linearly transformed to a 0–1 scale [58]. This approach helps to compare effect size and was validated in prior empirical research [37, 51, 59–61].

Furthermore, given the skewed distribution of e-cigarette use, we conducted additional sensitivity analyses with a log-transformed variable (log[x + 1]). Results were consistent with the main analyses (see Supplementary Figure S1). To address concerns about sample imbalance between LGB and non-LGB participants, we conducted a supplementary propensity score matching (PSM) analysis. Using 1:1 nearest-neighbor matching on demographic covariates, we obtained a matched sample of 149 LGB and 149 non-LGB participants. This procedure substantially improved covariate balance (all standardized mean differences < 0.1). Given the reduced sample size (~ 300), the PSM analyses were performed as robustness checks rather than the primary models.

## Results

### Preliminary analyses

The demographic features of the respondents were presented in Table 1. The average age of respondents is approximately 40 years. Overall, males accounted for 51.2% of the sample, slightly more than females at 48.8%. Most respondents (78.5%) had at least some college education. In terms of annual household income, about half (49.6%) fell within the range of ¥150,000 to ¥499,999. The majority of respondents (81.5%) reported living with a romantic partner.

Table 2 presents the descriptive statistics of the major variables along with the results of the zero-order correlation analysis. As shown, LGB identity was positively correlated with psychological distress. In addition, both psychological distress and traditional cigarette use were positively associated with e-cigarette use.

**Table 2:** Zero-order pearson correlations

Variables	Min	Max	Mean ± SD	1	2	3	4	5
1.Past 30-day e-cigarette use	0	30	1.27 ± 3.85	—	0.001	0.086**	0.044**	0.325**
2.Sexual Minority	0	1	0.03 ± 0.17		—	0.104**	− 0.014	− 0.027
3.Psychological distress	0	4	0.85 ± 0.81			—	− 0.041**	0.104**
4.eHealth use	0	9	6.33 ± 2.11				—	0.017
5. Past 30-day cigarette use	0	30	4.72 ± 8.34					—

a SD stands for standard deviation. *p <.05; **p <.01

### Testing mediation and moderation

H1 predicted a positive direct effect of sexual orientation on e-cigarette use. However, as shown in Table 3, the association did not meet the statistical threshold (*b*_*p*_ = 0.003, *β* = 0.057, *p* =.782), indicating a non-significant direct effect while controlling psychological distress, therefore H1 was not supported.

**Table 3:** Summary of mediation and moderation effects

Mediation pathway	bp	β	SE	95% CI	*P*
*a* path: SO→PSD	0.111	0.548	0.017	[0.077, 0.144]	< 0.001
*b* path: PSD→EC	0.038	0.062	0.009	[0.021, 0.055]	< 0.001
a*b path: SO→ PSD→EC	0.004	0.034	0.001	[0.002, 0.007]	
*d* path: SO→EC	0.003	0.057	0.010	[−0.017, 0.023]	0.782
Moderation pathway					
EH→*a* path	− 0.160	− 0.091	0.077	[−0.312, − 0.008]	< 0.05

All model controlling for gender, age, education, household income, partnership, and regular cigarette use

*bp* percentage coefficient, *β* standardized beta, *CI* confidence interval, *SO* sexual orientation, *PSD *psychological distress, *EC* e-cigarette use, *EH* eHealth use

**Fig. 2:**
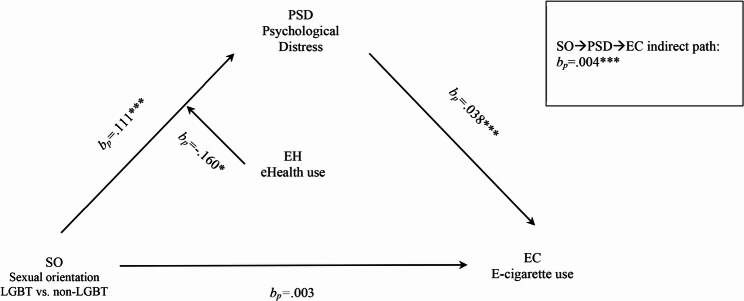
Results of moderated mediation model. Note. Path indicators are percentage coefficients, *b*_*p*_. *p<.05. **p<.01. ***p<.001

H2 predicted a positive mediation effect. Figure 2 revealed a positive indirect effect (*b*_*p*_ = 0.004, *β* = 0.034, bootstrap 95% CI ranges [0.002, 0.007], Sobel test *p* <.001), supporting H2. Specifically, being LGB was associated with higher psychological distress (*b*_*p*_ = 0.111, *β* = 0.548, *p* <.001), which in turn positive associated with e-cigarette use (*b*_*p*_ = 0.038, *β* = 0.062, *p* <.001). Interpreting *bp*, LGB individuals reported 11.1% higher psychological distress compared to heterosexual individuals.

Combining the results of H1 and H2, the findings indicate a full mediation effect [50], suggesting that the relationship between sexual orientation among Chinese adults and e-cigarette use is fully mediated by psychological distress.

H3 postulated a negative moderation effect of eHealth on the association between sexual orientation and psychological distress. Table 3 showed a negative moderation effect (*b*_*p*_ = − 0.160, *β* = − 0.091, *p* <.05), supporting H3. We further tested whether eHealth moderated the other relationships in the mediation model, but the results showed no significant effects.

**Fig. 3:**
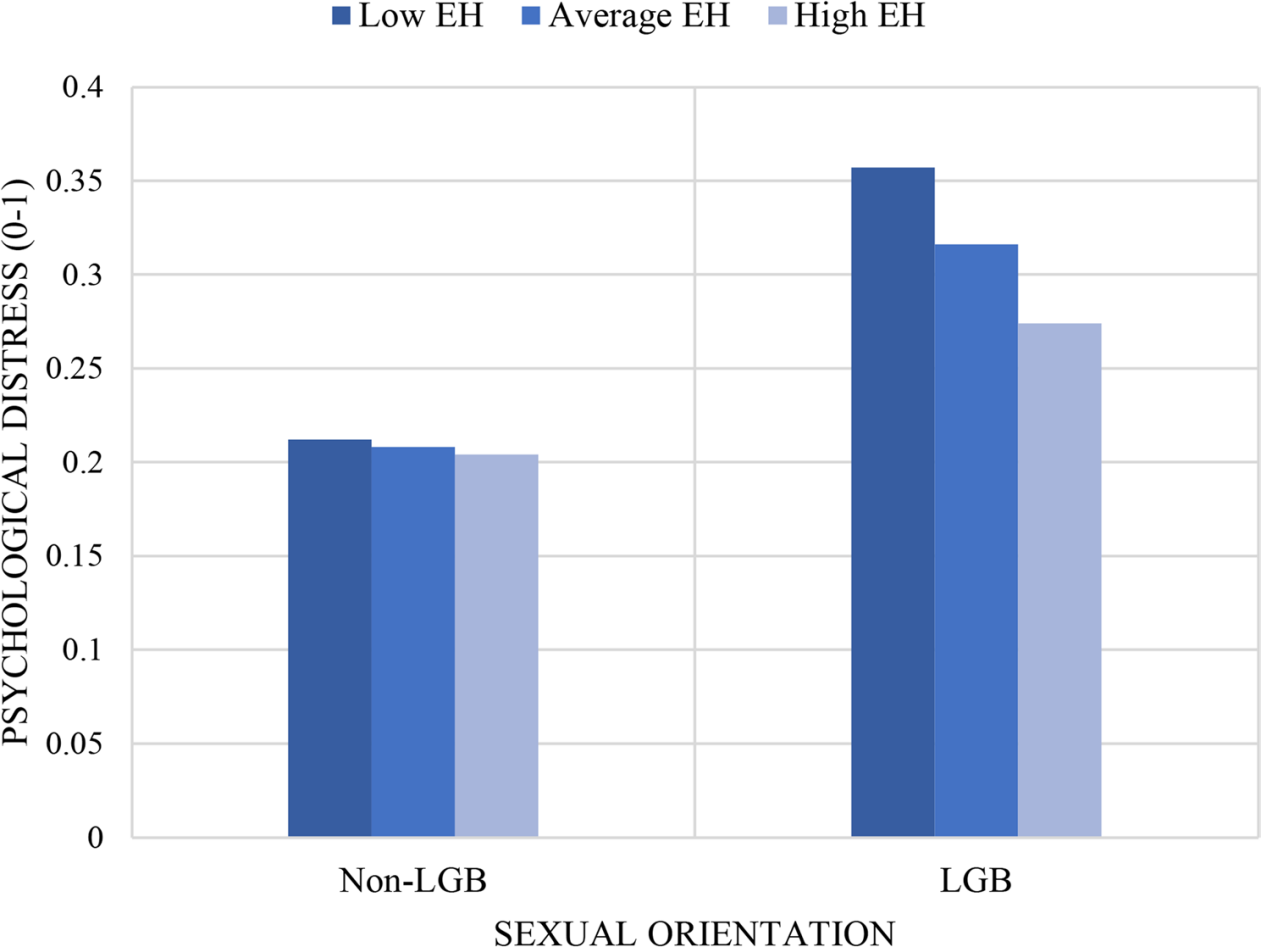
Moderation effect of eHealth use (EH)

Figure 3 visualizes the moderating effect. The figure illustrates that LGB participants consistently reported higher psychological distress than non-LGB participants across all levels of eHealth engagement. However, the magnitude of this disparity decreased as eHealth activities increased. At the low level of eHealth, the gap between LGB and non-LGB individuals was the largest, whereas at the high level of eHealth, the difference became smaller, indicating a buffering role of eHealth engagement for LGB group.

In the matched sample (*n* = 298), the mediation results were consistent with those obtained from the full sample, supporting the robustness of the main findings. However, the moderation effect was no longer statistically significant in the matched sample, which we attribute to limited statistical power given the smaller N. These supplementary results are reported in Supplementary Figure S2, which illustrates the mediation and moderation estimates in both the full sample and the PSM-matched sample.

## Discussion

Built upon the minority stress framework, this study is the first nationally representative study in China to investigate disparities in mental health and e-cigarette use between sexual minority and majority populations. The findings provide initial insights into the mental health status and e-cigarette usage patterns among Chinese LGB adults, contributing to existing literature. In addition to marketing strategies aimed at Chinese LGB adults, this population also exhibits a higher frequency of e-cigarette use compared to heterosexual adults, reflecting the usage of e-cigarette as a coping mechanism for psychological distress. While marketing may also influence the usage patterns, existing evidence suggests coping mechanism plays a more central role among Chinese LGB in managing prolonged distress as minority within a patriarchal culture [62, 63].

Consistent with prior findings [25–27, 35, 36], LGB individuals in China experience minority stress and report higher levels of psychological distress. Our study also identifies the mechanism linking sexual minority status to addictive behaviors [24, 33, 34]. Since the removal of homosexuality and bisexuality from the Chinese Classification of Mental Disorders in 2000 [64], social discrimination against LGB individuals has decreased significantly. However, the pressure to continue family lines in Chinese culture still contributes to invisible stress among Chinese LGB adults, leading them to use e-cigarettes as a coping mechanism for mental distress [1].

While research from some Western countries like the U.S. indicates that LGB individuals use e-cigarettes more than their heterosexual counterparts [16–18], this direct association is non-significant among Chinese LGB adults. This may be attributed to stricter e-cigarette marketing regulations implemented by the Chinese government [15] and the lower overall prevalence of e-cigarettes in China compared to the U.S [10]., resulting in weaker social norms of using e-cigarette.

Considering the significant indirect association and non-significant direct association between LGB identity and e-cigarette use, our study suggests a full mediation effect, which provides statistical evidence that sexual orientation in China influences e-cigarette use entirely through psychological distress. Therefore, the pattern of e-cigarette use among Chinese LGB individuals exhibits a unique characteristic: e-cigarettes primarily serve as a coping mechanism rather than being driven by commercial trends or social norms.

Furthermore, this study provides a novel finding of the moderating effect of eHealth use and its impacts on mitigating health disparities between LGB and heterosexual adults. Higher eHealth engagement was associated with smaller differences in psychological distress between LGB and heterosexual groups. Achieving health equality is an essential mission in public health [65, 66]. In the heterosexual society, eHealth could serve as a private, convenient, and cost-effective tool to lessen negative mental distress of being sexual minority populations [44, 46]. At the same time, our results showed that eHealth use did not moderate the relationship involving e-cigarette use, further supporting the notion that variations in e-cigarette use between LGB and heterosexual groups are primarily shaped by psychological status rather than by eHealth engagement [50].

The study has several implications. First, psychological distress significantly contributes to unhealthy behaviors among Chinese LGB individuals, such as e-cigarette use. Mental health professionals should prioritize addressing these issues and integrating tobacco prevention into clinical practices [51]. Second, given the current health disparities among Chinese sexual minority adults, public health policymakers should allocate resources to support their health needs. This could involve targeted educational campaigns to enhance health literacy and prevent addictive behaviors. Third, recognizing the effectiveness of eHealth in reducing health disparities, media planners and healthcare providers should utilize eHealth platforms to deliver psychological support to the LGB population [67].

### Limitations

We acknowledge several limitations in our study. First, it employed a cross-sectional survey design, which limits establishing causal relationships among sexual orientation, psychological distress, e-cigarette use, and eHealth use. Future research should consider longitudinal designs or experiments to validate causality. Second, the relatively small proportion of LGB participants (3%) may limit the statistical power for detecting more complex interaction effects and constrain the generalizability of our findings to the broader LGB population. Nevertheless, this proportion aligns with estimates in Chinses sample (around 5%) [68], lending some ecological validity to our sample. However, more complex moderated mediation effects should be interpreted with caution. Third, despite controlling for factors like traditional cigarette use, unmeasured variables could potentially influence the outcomes observed. Finally, the measure of eHealth use in this study was general and did not specifically focus on mental health or tobacco prevention. Future studies could explore the impacts of targeted digital mental health interventions.

## Conclusion

In summary, our study sheds light on the mechanism linking sexual orientation to e-cigarette use, mediated by psychological distress within the minority stress framework. The results reveal a positive indirect effect where sexual minority adults in China experience higher psychological distress, leading to increased e-cigarette use. This suggests that LGB individuals face greater psychological challenges and consequently use e-cigarettes more than heterosexual individuals. Furthermore, our findings underscore the moderating effect of eHealth use in reducing health disparities. Based on these insights, we recommend clinicians develop and advocate for eHealth interventions tailored to disadvantaged minority populations.

## Supplementary Information

Supplementary Material 1.

## Data Availability

Data will be made available on request.
