# Supplementary material for: Invisible minority stress and addictive behaviors: disparities in psychological distress and e-cigarette use among lesbian, gay, and bisexual adults in China
Source: BMC Psychol. 2026 Jan 17;14:311. doi: 10.1186/s40359-025-03875-7 (PMC12964716; doi:10.1186/s40359-025-03875-7)
Supplement: Supplementary file 1 — Supplementary Material 1. [file 40359_2025_3875_MOESM1_ESM.docx]

Figure S1. Results of moderated mediation model

SO

Sexual orientation

LGBT vs. non-LGBT

PSD

Psychological Distress

EC

E-cigarette use

EH

eHealth use

*b_p_=*.111***

*b_p_=*-.160*

*b_p_=*.001

*b_p_=*.014***

SO🡪PSD🡪EC indirect path:

*b_p_*=.002***

Note. E-cigarette use was log-transformed.

Figure S2. Results of moderated mediation model in the PSM-matched sample (n = 298)

SO

Sexual orientation

LGBT vs. non-LGBT

PSD

Psychological Distress

EC

E-cigarette use

EH

eHealth use

*b_p_=*.114***

*b_p_=*-.166

*b_p_=*.004

*b_p_=*.074*

SO🡪PSD🡪EC indirect path:

*b_p_*=.008***
